# Pyrazines Attract *Catocheilus* Thynnine Wasps

**DOI:** 10.3390/insects5020474

**Published:** 2014-06-19

**Authors:** Bjorn Bohman, Rod Peakall

**Affiliations:** 1 Research School of Chemistry, The Australian National University, Canberra ACT 0200, Australia; 2 Research School of Biology, The Australian National University, Canberra ACT 0200, Australia; E-Mail: rod.peakall@anu.edu.au; 3 School of Chemistry and Biochemistry, The University of Western Australia, Crawley WA 6009, Australia

**Keywords:** semiochemical, attractant, sexual deception, *Drakaea*, *Catocheilus*

## Abstract

Five previously identified semiochemicals from the sexually deceptive Western Australian hammer orchid *Drakaea livida*, all showing electrophysiological activity in gas chromatography–electroantennogram detection (EAD) studies, were tested in field bioassays as attractants for a *Catocheilus* thynnine wasp. Two of these compounds, (3,5,6-trimethylpyrazin-2-yl)methyl 3-methylbutanoate and 2-(3-methylbutyl)-3,5,6-trimethylpyrazine, were attractive to male wasps. Additionally, the semiochemical 3-(3-methylbutyl)-2,5-dimethylpyrazine, a close analogue to 2-(3-methylbutyl)-3,5,6-trimethylpyrazine, identified in five other species of thynnine wasps, was equally active. The three remaining compounds from *D. livida*, which were EAD-active against *Catocheilus*, did not attract the insects in field trials. It is interesting that two structurally similar compounds induce similar behaviours in field experiments, yet only one of these compounds is present in the orchid flower. Our findings suggest the possibility that despite the high specificity normally characterising sex pheromone systems, the evolution of sexual deception may not be entirely constrained by the need to precisely match the sex pheromone constituents and blends. Such evolutionary flexibility may be particularly important during the early stages of speciation.

## 1. Introduction

Orchids are well known to adopt fraudulent strategies to achieve pollination, with an estimated one third of the 25,000+ species believed to be pollinated by deception [1]. Among the most unusual of these deceptive pollination strategies is sexual deception, whereby male insects are attracted to flowers by semiochemicals that mimic the pheromones released by conspecific female insects during courtship [2]. Subsequently, pollination typically occurs when the male insect attempts copulation with the flower, a process known as pseudocopulation [3,4].

Several hundred Australian terrestrial orchids, representing multiple genera, secure pollination by the sexual deception of thynnine wasps [3,5,6]. Thynnine wasps represent a diverse and conspicuous component of the Australian insect fauna [7]. The parasitic female thynnines spend most of their lives underground, only appearing above ground to mate. Male thynnines make patrolling flights to locate the ‘calling’ females that perch on low vegetation where they release sex pheromones. Females are located rapidly, with the successful male picking up a female in flight before carrying her, *in copula*, to a nectar source for feeding [3]. 

The Australian terrestrial orchid genus *Drakaea* (hammer orchids), fully exploit the mating behaviour of thynnine wasps. The highly reduced flower consists of a hinged insectiform labellum (3rd modified orchid petal) of similar size to the flightless female wasp (see [3]). When a sexually attracted male wasp attempts to fly off with the labellum, the wasp is tipped upside down and brought into contact with the column where pollination occurs. Notwithstanding the remarkable morphological similarity between orchid and female wasp in this system, it has long been known that floral odours hold the key to long-range pollinator attraction [3]. Yet, we are only now beginning to understand the chemistry of sexual deception in this genus. 

The semiochemicals used by sexually deceptive orchids have only been confirmed for representatives of three genera: European *Ophrys* and Australian *Chiloglottis* and *Drakaea*. In the studied species of male bee pollinated *Ophrys*, sexual attraction is commonly achieved by complex mixtures of alkanes and alkenes, with species specificity achieved by unique blends of alkenes [2,8,9,10]. In *Chiloglottis* orchids specific thynnine wasp pollination is achieved by one, two or three components from a pool of six related compounds, all 2,5-dialkylcyclohexane-1,3-diones or ‘chiloglottones’ [5,11,12]. In *Drakaea* we recently discovered that pollination is mediated by pyrazine compounds (Table 1) [13,14]. For example, in *Drakaea glyptodon* (King-in-his-carriage orchid) a combination of different pyrazines, including a novel hydroxymethylpyrazine (2-hydroxymethyl-3,5-diethyl-6-methylpyrazine), functions as a semiochemical blend for sexually luring male *Zaspilothynnus trilobatus* to pollinate the flower [15]. These same compounds are also critical components of the sex pheromone of female *Z. trilobatus* wasps. Field bioassays further confirmed that a specific blend of these pyrazines is required to attract, initiate landing, and induce copulatory activity by the male wasp. Our discovery represented the first confirmed case of pyrazines as insect sex pheromones, and a new semiochemical system in plants, broadening our understanding of the chemical secrets of sexual deception and its evolution.

Here we build on our previous finding of tetrasubstituted pyrazines in the related warty hammer orchid *D. livida* [13]. This orchid is unusual among Australian sexually deceptive orchids, where pollination is most often highly specific [5], in that more than one pollinator species is exploited. Field observations combined with chemical analysis suggest the existence of two allopatric chemotypes, the first pollinated by *Zaspilothynnus nigripes* [14], the second by an undescribed species of *Catocheilus* [13,16]. However, when tested out of their natural geographic range, flowers from some sites have been observed to attract both pollinators, thus the relationship between the putative chemotypes and the two pollinators is not yet fully understood. This presence of chemotypic differences within *D. livida* may represent the early stages of speciation [13].

The goal of this present study was to evaluate the biological activity of six electrophysiologically active pyrazines, all strong candidates as orchid semiochemicals and/or sex pheromone components of thynnine wasps. The first five compounds were discovered in floral extracts of the *D. livida* putative chemotype that attracts *Catocheilus*. The sixth compound is reported here for the first time as a component of solvent extracts of wasp tissue and the headspace of ‘calling’ females from a number of thynnine species (see Table 1). The same compound has previously also been found in flowers of the distantly related dragon orchid *Caladenia barbarossa* [17].

Our specific aim was to determine which compounds are responsible for the attraction of *Catocheilus* wasps to *D. livida*, and to investigate the structural specificity and blends of compounds necessary for attraction.

## 2. Experimental Section

Details of plant and insect collection, and extraction and isolation protocols for identification, confirmation, and synthesis of all semiochemicals used, have been described previously [13,14,17]. 

### 2.1. GC-MS/EAD

GC-MS/EAD data were recorded using a HP GCD 1800A equipped with a BPX5 column (30 m × 0.25 mm × 0.25 μm film thickness, SGE Australia) using helium as a carrier gas. Synthetic samples were manually injected (1–3 µL of 0.1 µg/µL or 1.0 µg/µL solutions in dichloromethane). A GC effluent splitter was used to split the flow to the MS and EAD. The split for EAD was passed through a Syntech effluent conditioner [18] containing a heated transfer line, with the outlet placed in a purified and humidified airstream where the electrodes holding the antenna were presented. For each EAD run, a preparation of two antennae with their tips cut off and the connecting tissue in between the antennal bases still attached, was mounted on a holder consisting of two electrodes using electrode gel. The electrodes were connected to a PC via a Syntech Intelligent Data Acquisition Controller (IDAC2) for recording of EAD signals in GC-EAD/2011 [19]. 

**Table 1:** GC-EAD active pyrazines against male *Catocheilus* wasps; structures and presence in Western Australian sexually deceptive orchids and female thynnine wasps.

Group	Species	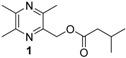	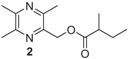	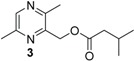	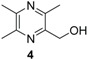	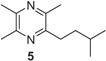	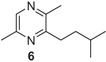
SEXUALLY DECEPTIVE ORCHIDS	*Drakaea livida*	x	x	x	x	x	
	*Drakaea thynniphila*				x		
	*Drakaea micrantha*				x		
	*Drakaea confluens*				x		
	*Caladenia barbarossa* [17]	x					x
THYNNINE WASPS	*Zaspilothynnus nigripes*						x
	*Zaspilothynnus gilesi*				x*		
	*Zaspilothynnus seductor*						x*
	*Zaspilothynnus rugicollis*						x*
	*Macrothynnus insignis*						x*

x = confirmed with co-injection with synthetic compound. x* = tentatively identified based on retention index and mass spectrum. Chemical names of compounds: 1 = (3,5,6-trimethylpyrazin-2-yl)methyl 3-methylbutanoate; 2 = (3,5,6-trimethylpyrazin-2-yl)methyl 2-methylbutanoate; 3 = (2,5-dimethylpyrazin-3-yl) 3-methylbutanoate; 4 = 2-hydroxymethyl-3,5,6-trimethylpyrazine; 5 = 2-(3-methylbutyl)-3,5,6-trimethylpyrazine; 6 = 3-(3-methylbutyl)-2,5-trimethylpyrazine.

### 2.2. Chemical Survey

The purpose of this survey was to screen additional sexually deceptive Western Australian *Drakaea* orchids and female thynnine wasps for compounds **1**–**6** (See Table 1) in order to evaluate their spread across related species. We used existing GC-MS data from previous studies [13,14,15,17] and also obtained additional species for analysis. In all cases, the screened floral extracts were obtained from solvent extracts made from multiple flowers, following a common protocol [15]. For the wasp survey, compounds were confirmed from at least two individual insects per species, either as head or body extracts or from headspace of sexually ‘calling’ females. Identifications of compound **6** from female *Zaspilothynnus gilesi*, *Z. seductor*, *Z. rugicollis and Macrothynnus insignis* are tentative, based on gas chromatography retention index and mass spectrum. No females of *Catocheilus* could be obtained, preventing their inclusion in this study.

### 2.3. Field Bioassays

Opportunities to conduct field bioassays are limited in this study system due to the narrow flight time of the pollinator (estimated to peak over 2–3 weeks for this species). Furthermore, male wasps search for mates during warm sunny periods, typically between 10 am and 2 pm, when temperatures exceed 18 °C. These constraints further reduce the number of suitable days available for conducting experimental bioassays in any one season, particularly in the study region where the weather conditions are highly variable during spring. Nevertheless, under suitable conditions, thynnine wasps will respond rapidly to appropriate synthetic samples, allowing bioassays to be conducted. Here we adapted the methods of Bohman *et al.* [15] to investigate the biological activity of compounds **1** to **6** (see Table 1). The synthetic compound(s) dissolved in dichloromethane were dispensed onto a dummy with the solvent allowed to evaporate before use. A solvent control was included in initial trials, confirming that the solvent was not attractive. The dummy consisted of a dressmakers pin, with a 4 mm diameter black plastic head, attached to a bamboo skewer, 25 cm above the ground (approximating the height of the orchid flowers). All bioassays were conducted during optimal weather conditions for wasp activity at the Ruabon Nature Reserve (WA) in October 2012. 

Because of the low amounts of compounds produced by female wasps and orchid flowers, it is presently not possible to reliably quantify the components of the headspace. Therefore, we do not know in advance either the optimal combination of compounds (noting GC-EAD activity does not necessarily equal biological activity), or their optimal ratios for stimulating a behavioural response. Therefore, as in Bohman *et al.* [15], we conducted preliminary bioassays with each compound (**1** to **6**) at the same concentration. By dispensing each compound onto individual beads, it was possible to test both the individual attractiveness of each compound, as well as various possible combinations by bringing two or more dummies together, such that the beads were virtually in contact. We also conducted choice tests among different sets of compounds, with two to four choices offered simultaneously. In these choice tests, the dummies were presented a minimum of 0.5 m apart, and perpendicular to the wind direction, in uniform habitat.

It was not feasible to test all possible combinations or concentrations of the semiochemicals. However, informed by the results from the closely related system *D. glyptodon*/*Z. trilobatus* [15], and by preliminary trials, we proceeded to implement a set of paired choice experiments for specific combinations and ratios that had been indicated to be attractive (See Figure 2). Each experiment commenced with preparation of the dummies that were loaded with 1–9 μL of different synthetic compounds/combinations based on working solutions of either 1 μg/µL or 10 µg/µL (see results for details). Note that the amount of compound applied (1 to a max of 30 µg) may at first seem to be high, given that the *Drakaea* flowers/female wasps produce such minute amounts of the compounds. However, only at these levels on the dummy can rates and intensity of male wasp attraction be achieved at the dummies that closely match those at orchid flowers (see [15]). Also, at these levels, pollinator responses to the bead can be maintained over the 1–2 h required for each experiment. Although not used for more than 2 h in formal experiments, dummies remain attractive for more than 24 h, indicating that the semiochemicals are released from the beads at very low levels, in a similar way to how they are released from flowers. A single experiment consisted of four to six trials of three min duration, with two choices presented simultaneously (>0.5 m apart, perpendicular to wind direction). Trials were abandoned if there was no response. The four main experiments were replicated twice, where possible on different days. Between trials, dummies were stored in a sealed container. *G*–tests applying William’s correction comparing the proportion of responses between the two choices for a given experiment were performed in GenAlEx 6.5 [20,21]. 

## 3. Results and Discussion

### 3.1. GC-MS/EAD

**Figure 1:**
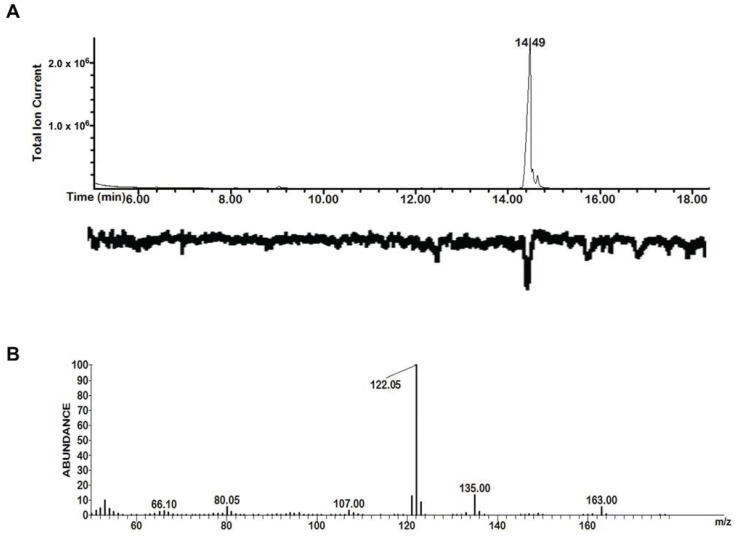
Total ion chromatogram/electroantennogram of 3-(3-methylbutyl)-2,5-dimethylpyrazine (**6**) on *Catocheilus* male wasp (**A**, retention time on top of peak) and the extracted mass spectrum (**B**, *m/z* on top of fragments).

Our study included the five compounds **1**–**5** from *Drakaea livida* (Table 1) previously identified as electrophysiologically active against *Catocheilus wasps* [13]. In addition we included 3-(3-methylbutyl)-2,5-dimethylpyrazine (**6**), which we reported earlier in the dragon orchid *Caladenia barbarossa* [17] (distantly related to *Drakaea*). Extended surveys, reported here for the first time, tentatively identified this compound **6** in solvent extracts from female thynnine wasps of four species, including *Z. nigripes*, one of the pollinators of *Drakaea livida*, and in the headspace of sexually 'calling' female wasps of other genera (Table 1). The finding that this alkylpyrazine is produced by females of multiple thynnine species, together with its structural similarity with compound **5** and similar biologically active alkylpyrazines in the *Drakaea glyptodon*/*Zaspilothynnus trilobatus* pollination system [15], prompted us to include compound **6** in this study. Furthermore, this compound was confirmed to be physiologically active by GC-MS/EAD against male *Catocheilus* (Figure 1, n = 10). The previously identified 2-hydroxymethyl-3-(3-methylbutyl)-5-methylpyrazine from *D. livida* pollinated by *Z. nigripes*, was shown not to be EAD-active on *Catocheilus*.

### 3.2. Chemical Survey

Our survey spanned three additional species of hammer orchids (*D. thynniphila, D. micrantha* and *D. confluens*), in which we previously have found pyrazines, and five species of thynnine wasps; *Zaspilothynnus nigripes*, *Z. gilesi*, *Z. seductor*, *Z. rugicollis* and *Macrothynnus insignis* (Table 1). Among the six compounds, only compounds **1**, **4**, and **6** were found in any of these other species. Compound **4** was found in flowers of *D. thynniphila*, *D. micrantha*, and *D. confluens*, as well as in females of *Z. gilesi*. Compounds **1** and **6**, as recently reported [17], were found in *C. barbarossa*. Compound **6** alone was found in four out of the five thynnine species examined: *Z. nigripes*, *Z. seductor*, *Z. rugicollis* and *M. insignis*, and has previously been found in plants such as stapiliads [22], and in a number of bacteria [23,24,25], phasmids [26], and ants [27,28,29,30,31]. 

### 3.3. Field Bioassays

Preliminary bioassays involving the presentation of compounds **1**–**6** as single compounds on their own, and in choice experiments (as single compounds spaced 0.5 m apart), revealed the compounds (3,5,6-trimethylpyrazin-2-yl)methyl 3-methylbutanoate (**1)**, 2-(3-methylbutyl)-3,5,6-trimethylpyrazine (**5**), and 3-(3-methylbutyl)-2,5-dimethylpyrazine (**6)** to be attractive to male *Catocheilus* wasps as single compounds. Further, preliminary trials involving various combinations of the six semiochemicals, where by multiple beads where positioned together with *ad hoc* compounds added or subtracted from the cluster of beads, failed to indicate biological activity of compounds **2**–**4**. Therefore, we focussed the additional bioassays reported below on these three semiochemicals (**1**, **5** and **6**). 

It is of interest that compounds **1** and **5** consistently showed the strongest EAD-activity in the *Catocheilus* wasps out of the five compounds originally identified from *Drakaea livida* (see Figure 1 in [13]). Compound **6** was also revealed to exhibit strong EAD-activity in this current study (Figure 1). Nevertheless, we acknowledge that our preliminary trials could not comprehensively test all possibilities given the limited opportunities available to conduct experiments. Therefore we do not rule out biological activity for the other compounds in untested combinations. However, the finding that the GC-EAD active compounds **2**–**4** were not even weakly attractive as single compounds contrasts with the case of *Drakaea glyptodon/Zaspilothynnus trilobatus*, where all five GC-EAD active compounds attract the pollinators in the field, although only one alkylpyrazine and one hydroxymethylpyrazine at a specific ratio (3:1) are critical for stimulating copulatory behaviour.

**Figure 2:**
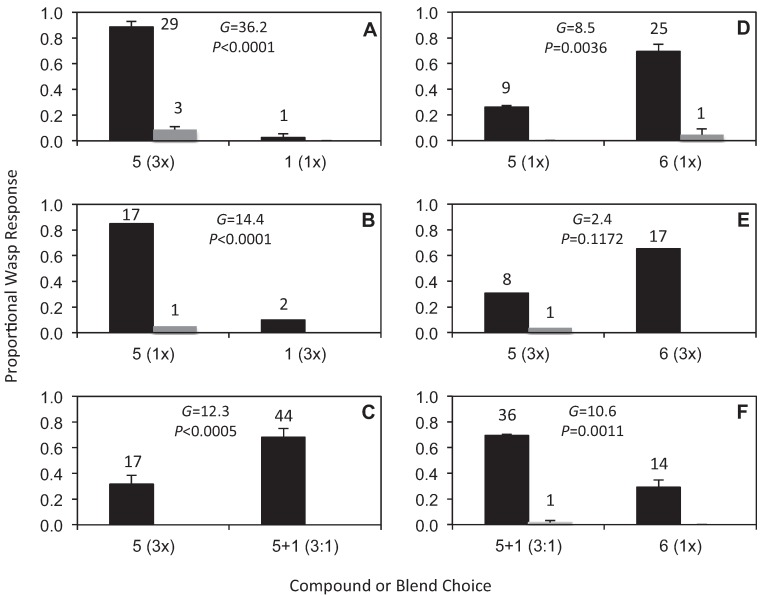
Responses of male *Catocheilus* wasps to bioassays presenting choices of compounds **1**, **5** and **6 **in different combinations and at different concentrations. The wasp responses are shown as proportions of the total, further partitioned into *approach* (black) *versus* combined *land* and *attempted copulation* (grey). The number of wasps responding to each treatment and the outcomes of *G*-tests for the null hypothesis of no difference between the two choices, based on the total number of responses, are also shown. (**A**) Compounds **1** *versus* **5** with **5** at 3-fold higher concentration. (**B**) Compounds **1** *versus* **5** with **1** at 3-fold higher concentration. (**C**) Compound **5** *versus* a blend of **5** and **1** at a 3:1 ratio. (**D**) Compound **5** *versus* **6** at equal 1-fold concentration (1×). (**E**) Compound **5** *versus* **6** at equal 3-fold higher concentration (3×). (**F**) Compound **6** *versus* a blend of **5** and **1** at a 3:1 ratio. For experiments **A**, **C**, **D** and **F**, proportions are shown as means ±se, each calculated across two replicate experiments. For the 1× concentration 3 µL of a 10 μg/μL solution of the compound or blend was applied to the bead and allowed to evaporate before use.

Figure 2 demonstrates the outcomes of six bioassay experiments with wasp responses shown as mean proportions of the total, further partitioned into *approach versus* combined *land* and *attempted copulation*. As in our previous studies (e.g., [5,15]), we present responses as proportions, since the use of proportions takes into account any variation in the total number of wasps responding, and allows for more meaningful comparisons across the experiments. Across the bioassays, a total of 226 wasp responses were scored, with the number of wasps responding to each treatment overlaid on the graphs. The outcomes of *G*-tests for the null hypothesis of no difference between the two choices, based on the total number of responses, are also shown (Figure 2). In all but one case a significant difference between the paired treatments was detected. Compound **5** was strongly attractive when presented at 3x the concentration of **1** (Figure 2A, 3× **5** *versus* 1× **1**, across 2 reps), with the three lands/attempted copulations only observed at **5**. A similar strong response to **5** but very weak response to **1** was also found when the concentration of the two compounds was reversed (Figure 2B, 1× **5** *versus* 3× **1** across 1 rep). Furthermore, no significant difference was detected between these two experiments (*G* = 0.924, *DF* = 1, *p* = 0.337, *N = 63*; *G*-tests applying William’s correction comparing wasp responses to compounds **1** and **5** across reversed concentrations). Thus compared to **1**, compound **5** is strongly attractive irrespective of concentration differences. However, when compound **5** was compared with a blend of compounds **5** and **1** (at a 3:1 ratio), the blend was significantly more attractive than compound **5** alone (Figure 2C, 3× **5** *versus* **5**+**1** at 3:1, across 2 reps). The 3:1 ratio was chosen for these tests *a priori* to match the optimal ratio of alkylpyrazine to hydroxymethylpyrazine known to be effective in pollinator attraction in *Drakaea glyptodon* [15].

An unexpected discovery was that compound **6** (not found in orchid flowers), was significantly more attractive than compound **5** (Figure 2D, 1× **5** *versus* 1× **6**, across 2 reps). Although not significant, the trend of a stronger response to **6**, with a weaker response to **5**, was also found when the concentrations of the two compounds were both increased 3-fold (Figure 2E, 3× **5** *versus* 3× **6**, across 1 rep). No significant difference was detected between these two experiments, confirming a common pattern of response (*G* = 0.547, *DF* = 1, *p* = 0.459, *N = 63*; *G*-tests applying William’s correction comparing wasp responses to compounds **5** and **6** at 1- and 3-fold concentrations). However, when compound **6** was compared with a blend of compounds **5** and **1** (at a 3:1 ratio), the blend was again significantly more attractive than a single compound (Figure 2F, **5 **+ **1** at 3:1 *versus* 1× **6**, across 2 reps). While there was no opportunity to comprehensively test other blends of **5** and **1** at different ratios, preliminary trials at other ratios continued to indicate that blends of these two compounds were more attractive than single compounds. 

The structures of compounds **5** and **6** are very similar, with the only difference being the presence or absence of a methyl group on the pyrazine ring. Nevertheless, the *Catocheilus* wasps demonstrated a significant preference for **6** over **5**, when only these choices were offered. By contrast, as already noted **6** was less attractive to a blend of **5** and **1** (at 3:1 ratio) (Figure 2). It is also noteworthy that the three additional pyrazine compounds **2**–**4**, all EAD-active against the male wasp, were not attractive in field experiments despite the structural similarity with compound **1**. No repellent effect was observed from any of the EAD-active compounds (**2**–**4)**, so the function of these compounds remains unknown. Comparing amplitudes of the signals in the EAD analyses [13] shows that the signals for **1** and **5** are the strongest, so it is possible that the remaining compounds are simply analogues, which elicit moderate EAD responses, but not behavioural activity in the natural context of field bioassays. In the future it will be of interest to investigate additional combinations of compounds **1** and **6** with the carboxylic esters **2** and **3**, as the latter compounds are structurally closely related to compound **5**. Unfortunately, due to the unusual seasonal conditions during the spring of 2013, which was one of the wettest on record at the field site, we were unable to perform any additional experiments with this species. Compound **5** was chosen rather than **2** or **3** in the 2012 season because the EAD response was stronger, and because this compound was, although weakly, still attractive on its own in field tests. It will also be of interest to determine whether the combination of an alkylpyrazine and a hydroxymethylpyrazine, as found in the case of *D. glyptodon*/*Z. trilobatus* [15] and this present study, is a common feature. Structure-activity studies may also help us better understand the basis of the strong specificity that typically characterises sexual deception in *Ophrys* [2,10,32], *Chiloglottis* [5,11,12] and *Drakaea* [16]. 

### 3.4. Evolutionary Implications

In this study we have extended our understanding of how male wasp attraction is achieved in a species belonging to the genus *Catocheilus*. This is only the second study where pyrazines are confirmed by field bioassays to be involved in the attraction of male thynnine wasps. In the first case to confirm pyrazines as sex pheromone components, we showed that in *Zaspilothynnus trilobatus* three alkylpyrazines and one novel hydroxymethylpyrazine elicit strong sexual attraction of males. These same semiochemicals are used by *D. glyptodon* to sexually lure *Z. trilobatus* males to the flower. In bioassays with semiochemicals, and at *D. glyptodon* flowers, it is typical that only a proportion of *Z. trilobatus* males land, with even fewer attempting copulation [3,15]. In the case of response to flowers, the combined lands/attempted copulations represented 24% of the total wasp responses. At the dummies, the combined lands/attempted copulations varied with the treatment from 0% to 45% [15]. 

In the present study of *Catocheilus*, combined lands/attempted copulations varied with the treatment from 0% to 9% (Figure 2), but did not reach the higher levels of sexual stimulation observed in the case of *Z. trilobatus*. This result may suggest that we either have not found all components of the sex pheromone, or that we have yet to empirically determine the optimal blend ratios of the sex pheromone components. In all thynnine wasp genera the females are difficult to locate since they spend most of their lives underground, only emerging to mate and feed for a short period of time. Furthermore, in cases such as the studied *Catocheilus*, which appear not to feed on flowers during mating, females are particularly difficult to acquire. In this present case, the identity of the pheromone remains unknown since no female *Catocheilus* have ever been located. Thus, for now we are reliant on the orchid, which sexually lures the male of this wasp species, to unravel clues about the sex pheromone.

Other studies of the chemical basis of sexual attraction in sexually deceptive orchids have found that the biologically active compounds emitted by the orchids are identical to constituents of the pollinators’ sex pheromone (e.g., *Chiloglottis trapeziformis* [11]; *Drakaea glyptodon* [15] and *Ophrys iricolor* [9]). Stökl *et al.* have also previously shown that *Ophrys* species sharing the same pollinator—independent of their phylogenetic relationship—use the same volatiles for pollinator attraction. In the case of the *Ophrys fusca* group, differences between the species mainly involved quantitative differences between the same compounds [33]. In *Ophrys exaltata* the bee pollinator *Colletes cunicularius* prefers the floral odour of the orchid mimic over the sex pheromone, a behaviour that has been explained as receiver bias towards novel signals [34].

In our study, a discovery of interest was the substitutability of the two alkylpyrazines **5** and **6**. For example, compound **6** (not found in the orchid flowers) was more attractive than compound **5** (found in the orchid), while a 3:1 blend of compounds **5** and **1** was more attractive than compound **6** on its own. Furthermore, compound **6** has been repeatedly detected in headspace extracts of female thynnine wasps, but as yet has not been found in *Drakaea* orchid flowers. This finding may suggest that these orchids lack the biochemical capability to synthesise compound **6**, and instead achieve equivalent sexual attraction by substituting this wasp-produced compound with a specific blend of orchid analogues. If we could confirm this hypothesis by further research, it would suggest a strong potential for evolutionary flexibility, despite the high specificity normally characterising sex pheromone systems (and the orchid pollination systems that have co-opted these same chemical systems). Thus the evolution of sexual deception may not be as tightly constrained by the need to precisely match the sex pheromone constituents and blends of the pollinator as it would seem at first. Furthermore, such flexibility may have been particularly crucial during the early stages of the evolution of sexual deception, as well as in the early stages of pollinator switching, which is known to be frequently associated with speciation in some sexually deceptive orchids [5]. 

## 4. Conclusions

Male *Catocheilus* wasps are strongly attracted to a blend of the floral volatiles (3,5,6-trimethylpyrazin-2-yl)methyl 3-methylbutanoate (**1)** and 2-(3-methylbutyl)-3,5,6-trimethylpyrazine (**5**) of the hammer orchid *Drakaea livida*. The wasp semiochemical 3-(3-methylbutyl)-2,5-dimethylpyrazine (**6**), found in other wasp species of the genera *Zaspilothynnus* and *Macrothynnus*, can be considered a structural analogue to **5**, and was also found to be an effective attractant to the male *Catocheilus* wasps. Structure-activity studies on floral semiochemicals and wasp sex pheromones are now required to gain a broader understanding of the specificity of chemicals mediating sexual deception and opportunities for evolutionary flexibility. 
